# CD80 (B7-1) as a potential therapeutic target in Epstein–Barr virus‑associated B cell diseases

**DOI:** 10.1038/s41598-026-55043-5

**Published:** 2026-05-26

**Authors:** Rin Yoshizato, Mariko Miura, Yumi Tamura, Yohei Kawano, Yuya Hokama, Nanami Soda, Yusei Ota, Kotomi Yamashita, Taisei Fukushima, Masataka Ishimura, Shouichi Ohga, Tomoharu Yasuda

**Affiliations:** 1 Department of Immunology, Graduate School of Biomedical and Health Sciences, Hiroshima University, Hiroshima, Japan; 2 Department of Pediatrics, Graduate School of Medical Sciences, Kyushu University, Fukuoka, Japan; 3 Present Address: Department of Immunology, Ehime University Graduate School of Medicine, Ehime, Japan

**Keywords:** Cancer, Immunology

## Abstract

Epstein-Barr virus (EBV)-associated B-cell lymphomas pose a therapeutic challenge due to their resistance to conventional chemotherapy and limited efficacy of current targeted approaches. Here, we identified CD80 as a cell surface antigen highly expressed in EBV-positive B-cell lymphomas. Monoclonal antibodies were generated from mice immunized with recombinant human CD80 and subsequently engineered into chimeric formats. These antibodies were evaluated for their cytotoxic activity against CD80-expressing lymphoma cells. Notably, high-affinity clones elicited robust antibody-dependent cellular cytotoxicity (ADCC) but lacked complement-dependent cytotoxicity (CDC), in contrast to rituximab, which targets CD20 and mediates both ADCC and CDC. Our findings suggest that cytotoxic effector functions are regulated not only by the constant region of the antibody but also by the antigen recognized by its variable region. Importantly, these CD80-specific antibodies do not inhibit T cell responses against EBV-positive immortalized cells, highlighting their potential as promising therapeutic candidates for EBV-positive lymphomas and other CD80-expressing malignancies.

**Supplementary Information:**

The online version contains supplementary material available at 10.1038/s41598-026-55043-5.

## Introduction

Epstein-Barr virus (EBV) is one of the most prevalent human herpesviruses in the world, infecting over 95% of the adult population^1^. Following primary infection, typically asymptomatic in childhood, EBV establishes lifelong latency by anchoring its genome within the nuclei of infected B cells. This latent state allows the virus to persist without active replication, effectively evading immune surveillance^2^.

While EBV infection is usually well controlled by the host immune system, it can lead to a range of diseases in both immunocompromised and immunocompetent individuals. These include infectious mononucleosis, Burkitt lymphoma (BL), Hodgkin lymphoma (HL), chronic active EBV disease (CAEBV), EBV-associated lymphoproliferative disorder (LPD), nasopharyngeal carcinoma, and gastric cancer^1,3^. In addition, EBV has been implicated in the pathogenesis of several autoimmune diseases, such as multiple sclerosis (MS), systemic lupus erythematosus (SLE), and rheumatoid arthritis (RA)^2^.

Among EBV-associated malignancies, diffuse large B-cell lymphoma (DLBCL) is particularly notable. EBV-positive DLBCL has been associated with poorer clinical outcomes compared to EBV-negative cases^4–6^. Similarly, EBV positivity correlates with reduced overall and disease-specific survival in HL^7^. In contrast, sporadic BL does not appear to show prognostic differences based on EBV status^8^.

CD80, also known as B7-1, is a protein belonging to the immunoglobulin superfamily and is expressed on the surface of antigen-presenting cells (APCs)^9^. It binds to CD28 on T cells and transmits activation signals, playing a critical role in T cell activation^10^. CD80 has been recognized for its dual function in both immune activation and tolerance. In addition to promoting T cell activation, CD80 interacts with CTLA-4, a negative regulator on T cells. CTLA-4 competes with CD28 for binding to CD80, thereby suppressing T cell activation^11^. Through these interactions, CD80 is involved in various immune responses and has been implicated in the development and progression of autoimmune diseases, allergic diseases, and cancer^9^.

Treatment of EBV-associated lymphomas remains challenging due to resistance to conventional chemotherapy and limited efficacy of current targeted therapies^12^. CD20-targeted agents like rituximab are often insufficient, especially in immunocompromised patients such as those with AIDS or post-transplant, where EBV reactivation is difficult to control^13–15^. Additionally, the immunosuppressive environment complicates therapeutic strategies, requiring a delicate balance between restoring immune function and suppressing tumor growth.

In this study, we identified CD80 as a cell surface antigen highly expressed on EBV-positive B-cell lymphoma. To clarify whether antibodies targeting CD80 exhibit specific cytotoxic activity against EBV-positive lymphoma cells, we isolated monoclonal antibodies (mAbs) from mice immunized with human CD80 (hCD80) and produced chimeric antibodies engineered with human IgG1 or IgG3 Fc. These antibodies were then evaluated for their cytotoxic activity against CD80 expressing lymphoma cells.

## Results

### Expression levels of CD80 in EBV-positive lymphoma cells and transformed B cells

**Fig. 1:**
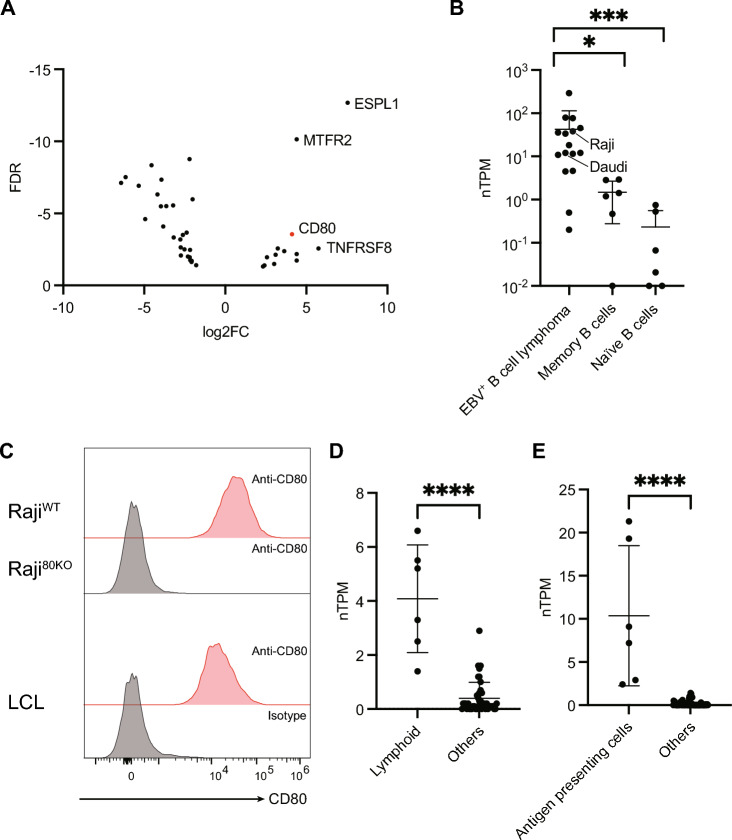
Transcriptome analysis reveals CD80 expression in EBV-infected cells (**A**) Volcano plots of the differentially expressed genes (DEGs) identified from the Human Protein Atlas (HPA) datasets. Normalized transcripts per million (nTPM) from 16 EBV-positive B cell lymphoma datasets were compared with nTPM of 12 normal B cell datasets (6 memory B cells and 6 naïve B cells). Membrane gene was selected using the HPA datasets. FDR, False discovery rate. (**B**) CD80 expression levels in 16 EBV-positive cell line datasets and 12 normal B cell datasets (6 memory B cells and 6 naïve B cells). A constant of 0.01 was added to handle zero values. EBV^+^, EBV-positive. (**C**) Representative histograms of CD80 expression on RajiWT, Raji80KO and lymphoblastoid cell lines (LCLs) established from PBMCs of healthy donors in our laboratory. RajiWT, wild-type Raji cells; Raji80KO, CD80-knockout Raji cells. (**D**) CD80 expression levels in normal tissue from the HPA datasets. Tonsil, lymph node, appendix, spleen, thymus, and bone marrow are defined as lymphoid organs. (**E**) CD80 expression levels in single-cell types in the HPA datasets. Langerhans cells, monocytes, B cells, macrophages, Kupffer cells, and Hofbauer cells are classified as antigen presenting cells. Data are presented as mean ± SD. Statistical significance was determined using the Mann-Whitney *U* test with exact *P* values or Dunn’s multiple comparisons test. *P* values are indicated as **p < 0.01; ***p < 0.001; ****p < 0.0001. ns, not significant.

To search for membrane proteins specific to EBV-positive B-cell lymphoma, EBV-positive B-cell lymphoma and normal B cells in the human protein atlas (HPA) data set were compared. After excluding genes widely expressed in normal tissues and brain, *CD80*, *ESPL1*, *MTFR2*, and *TNFRSF8* (CD30) were extracted as genes significantly upregulated in EBV-positive B-cell lymphomas (Fig. 1A). *ESPL1* and *MTFR2* were excluded from candidate targets due to their predominant localization in the nucleoplasm and vesicles, respectively. Monoclonal antibodies targeting CD30 have already been clinically applied in the treatment of HL and DLBCL using brentuximab vedotin, an antibody-drug conjugate containing monomethyl auristatin E^16,17^. In this study, we focused on CD80, which has not yet received attention as a target for EBV positive lymphomas. Expression of *CD80* was significantly increased in EBV-positive B-lymphoma cells compared to normal B cells (Fig. 1B) and EBV-negative B-lymphoma cells (Supple Fig. 1A). Next, to evaluate the safety of CD80 as a therapeutic target, its expression levels in normal tissues were assessed. Surface staining of CD80 in Raji cells, an EBV-positive human BL cell line, showed high level of signals which was abrogated by the CRISPR-Cas9-mediated knockout of *CD80* gene (Fig. 1C). CD80 was also highly expressed in EBV-transformed human lymphoblastoid cell lines (LCLs) (Fig. 1C). Analysis of public data from HPA revealed that expression of *CD80* is highly specific to lymphoid tissues compared to non-lymphoid tissues (Fig. 1 D, Supple Fig. 1B). Furthermore, a comparison of cell types expressing *CD80* revealed that it is highly expressed in antigen presenting cells such as Langerhans cells, monocytes, B cells, and macrophages (Fig. 1 E, Supple Fig. 1C). These findings suggest that targeting CD80 may affect the antigen presenting function of the immune cells, but with minimal risk of off-target side effects.

### Established anti-CD80 mAbs from hCD80-binding mouse single memory B cells

**Fig. 2:**
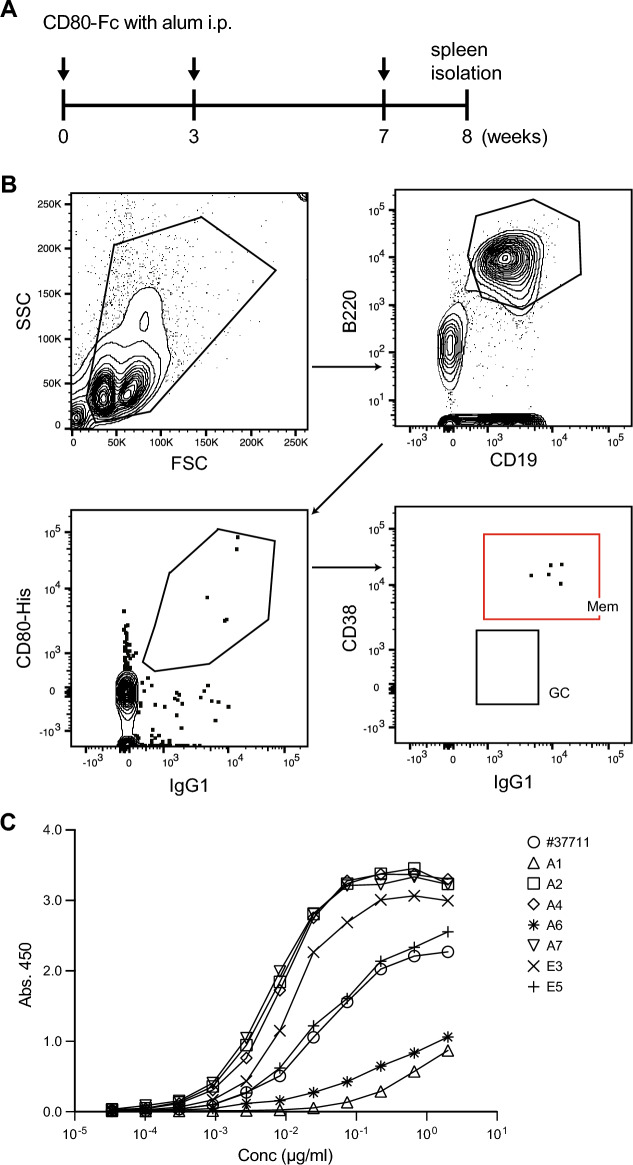
Anti-CD80 mAbs established from antigen-specific single memory B cells (**A**) Schematic diagram of the experimental design. C57BL/6 mice were immunized at weeks 0, 3, and 7. Each immunization consisted of alum-precipitated 100 µg CD80-Fc. Mice were analyzed one week after the final immunization. (**B**) Gating strategy for FACS sorting of antigen-specific memory B cells. Lymphocytes were gated by forward and side scatter. B220^+^CD19^+^IgG1^+^CD38^+^His-tag^+^ live memory B cells were sorted. (**C**) ELISA analysis of CD80-specific mAbs. Clone #37711 (R&D Systems, #MAB140-SP) used as a positive control. CD80-binding activity was measured by ELISA using plates coated with CD80-His.

To generate hCD80-specific mAbs, mice were intraperitoneally immunized with alum-precipitated recombinant hCD80-Fc fusion proteins. Immunization was performed three times in total, and spleens were collected one week after the final immunization (Fig. 2A). CD80 extracellular domain (CD80ECD)-specific memory B cells (CD19^+^B220^+^IgG1^+^CD38^+^His-tag^+^) were detected and single-cell sorted by flow cytometry (Fig. 2B). The heavy chain (HC) and light chain (LC) genes were amplified by PCR from sorted single cells as previously described^18,19^. Of the twenty antigen-specific memory B cells, HC genes were successfully amplified in 16 cells (80%), and LC genes in 18 cells (90%). Expression vectors for HC and LC were then co-transfected into Expi293F cells. ELISA with culture supernatants showed that seven clones had the ability to bind to CD80-His (Fig. 2C).

**Fig. 3:**
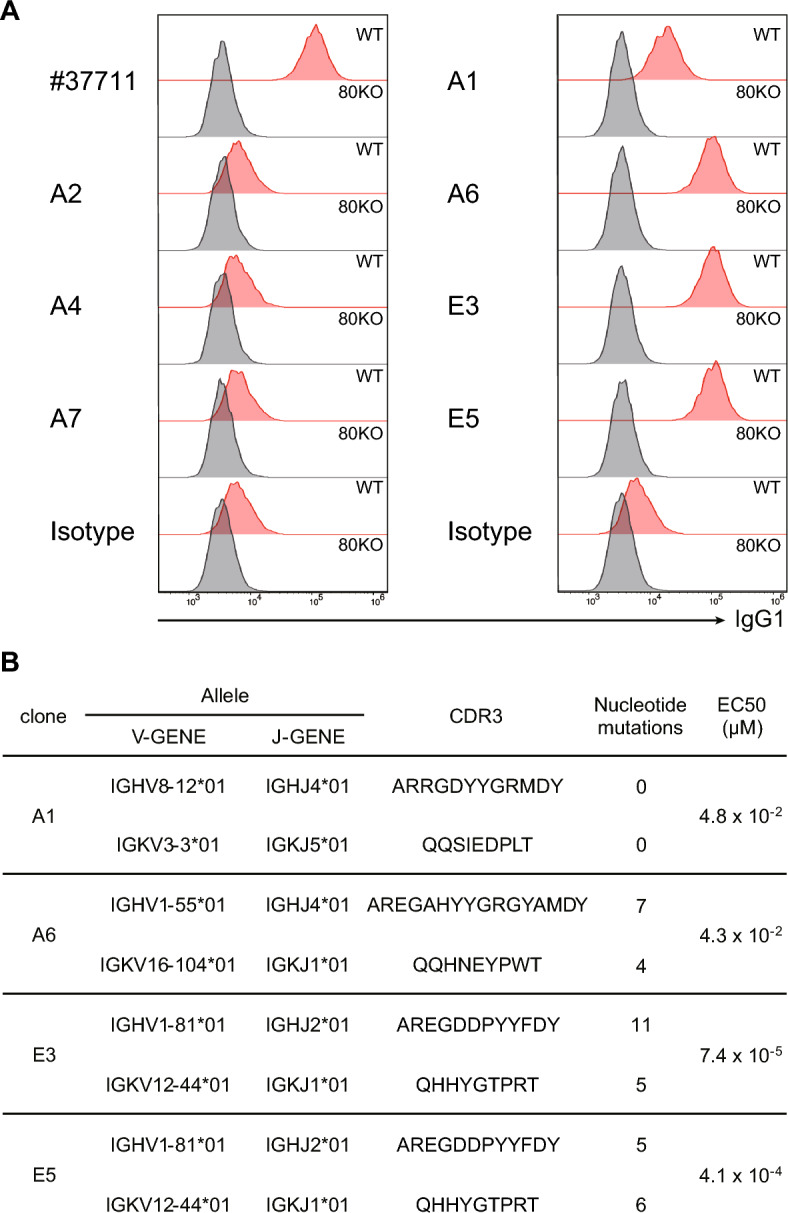
Establishment of antibodies with multiple somatic hypermutations binding to cell surface CD80 (**A**) Evaluation of CD80-specific antibody binding to the cell surface. Clone #37711 (R&D Systems, #MAB140-SP) used as a positive control. Mouse IgG1 UNLB (SouthernBiotech, #0102-01) was used as an isotype control (Isotype). FITC anti-mouse IgG1 (BioLegend, #406605) was used as the secondary antibody. WT, wild-type Raji cells; 80KO, CD80-knockout Raji cells. (**B**) VH and VL genes, CDR3 amino acid sequence, number of nucleotide mutations, and EC_50_ values for each CD80-specific mAb clones are shown. EC_50_ values were calculated from the same dataset as in Fig. 2C using nonlinear regression curve fit on Prism software.

Next, to evaluate the specific binding ability to cell surface CD80, screening was conducted using wild-type Raji cells (Raji^WT^) and CD80 knockout Raji cells (Raji^80KO^) with a commercially available anti-CD80 antibody (#37711) as the positive control. Among the seven CD80-specific mAbs, four clones were confirmed to specifically recognize CD80 on the cell surface (Fig. 3A). Sequence analysis using IMGT/V-quest revealed that clone A1 had no somatic hypermutation (SHM). Clones E3 and E5 shared identical V and J genes as well as CDR3 sequences, suggesting that they originated from a common ancestral germinal center B cell (Fig. 3B). Three clones, A6, E3, and E5, exhibited multiple SHMs and were presumed to have undergone affinity maturation, therefore, they were selected for downstream analysis.

### Anti-CD80 mAbs exhibit ADCC activity but lack CDC function

Chimeric antibodies were generated by fusing the constant regions of anti-hCD80 mouse clones (A6, E3, and E6) with human IgG1 (IGHG1*08) or IgG3 (IGHG3*01), and their binding specificity was subsequently confirmed (Supple Fig. 2A, B). To enhance affinity for FcγRIIIa, three amino acid substitutions (S239D, A330L, I332E) were introduced into the human IgG3 Fc domain, resulting in the IgG3^DLE^ variant^20^. IgG1-type mAbs (A6G1*08, E3G1*08, and E5G1*08) bound specifically and with high affinity to monomeric CD80-His, with dissociation constants (K_D_) of 14.5 nM, 0.14 nM, and 0.96 nM, respectively (Supple Fig. 3).

**Fig. 4:**
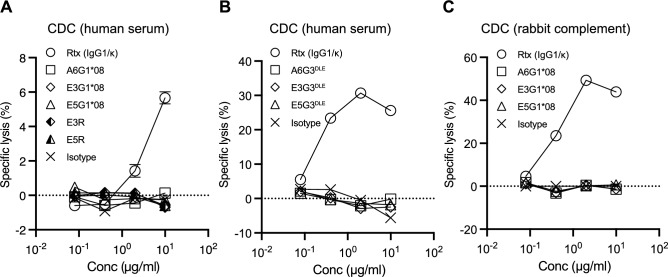
CDC assay with Raji cells (**A**) CDC assay using human serum and Raji^WT^ cells. IGHG1*08-type (A6G1*08, E3G1*08 and E5G1*08) and rituximab-type (E3R, E5R) chimeric antibodies were used at the indicated concentrations. Rtx, rituximab; std, Human IgG1 Kappa-UNLB (SouthernBiotech, # 0151K-01). (**B**) CDC assay using human serum and Raji^WT^ cells. IgG3^DLE^-type (A6G3^DLE^, E3G3^DLE^ and E5G3^DLE^) chimeric antibodies were used at the indicated concentrations. Rtx, rituximab; std, Human IgG3 Lambda-UNLB (SouthernBiotech, # 0153L-01). (**C**) CDC assay using rabbit complement and Raji^WT^ cells. IGHG1*08-type (A6G1*08, E3G1*08 and E5G1*08) chimeric antibodies were used at the indicated concentrations. Rtx, rituximab; Isotype, Human IgG1 Kappa-UNLB (SouthernBiotech, # 0151K-01). Data represent two independent experiments and are shown as the mean of duplicate technical measurements with error bars indicating the range.

First, we evaluated complement-dependent cytotoxicity (CDC) against Raji^WT^ cells. As previously reported^21^, CD20-specific chimeric mAb, rituximab (Rtx, IgG1/κ) exhibited CDC activity in both human serum (Fig. 4A and 4B) and rabbit complement (Fig. 4C). In contrast, none of the CD80-specific IgG1 or IgG3 mAbs showed CDC activity (Fig. 4A-C). The difference in the amino acid sequence of the CH1 constant region between Rtx IgG1 and IgG1*08 may be critical for CDC activity. We substituted the amino acids in E3G1*08 and E5G1*08 with the Rtx-type (R214K and V215A), however, the CDC activity of these modified anti-CD80 IgG1 (E3R and E5R) remained at baseline levels (Fig. 4A).

**Fig. 5:**
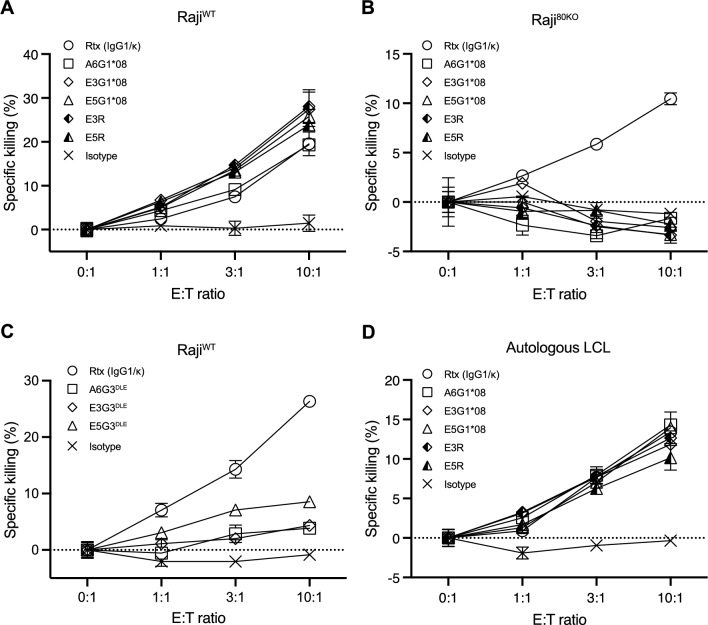
ADCC assay with EBV-infected cell lines (**A**-**D**) NK cell-mediated specific killing at the indicated effector-to-target (E:T) ratios against Raji^WT^ (**A**, **C**), Raji^80KO^ (**B**), and autologous LCLs (**D**) using IGHG1*08-type (A6G1*08, E3G1*08 and E5G1*08), rituximab-type (E3R, E5R), and IgG3^DLE^-type (A6G3^DLE^, E3G3^DLE^ and E5G3^DLE^) chimeric antibodies. Rtx, rituximab; Isotype, Human IgG1 Kappa-UNLB (SouthernBiotech, # 0151K-01) (**A**, **B**, **D**) or Human IgG3 Lambda-UNLB (SouthernBiotech, # 0153L-01) (**C**). Data represent two independent experiments and are shown as the mean of duplicate technical measurements with error bars indicating the range.

Conversely, evaluation of ADCC using Raji^WT^ and Raji^80KO^ cells confirmed that all three IgG1 mAbs (A6G1*08, E3G1*08, and E5G1*08), which specifically target CD80, exhibited ADCC activity (Fig. 5A and 5B; Supple Fig. 4). Galiximab, a previously reported CD80 antibody, also demonstrated ADCC activity against Raji^WT^ cells (Supple Fig. 5).

**Fig. 6:**
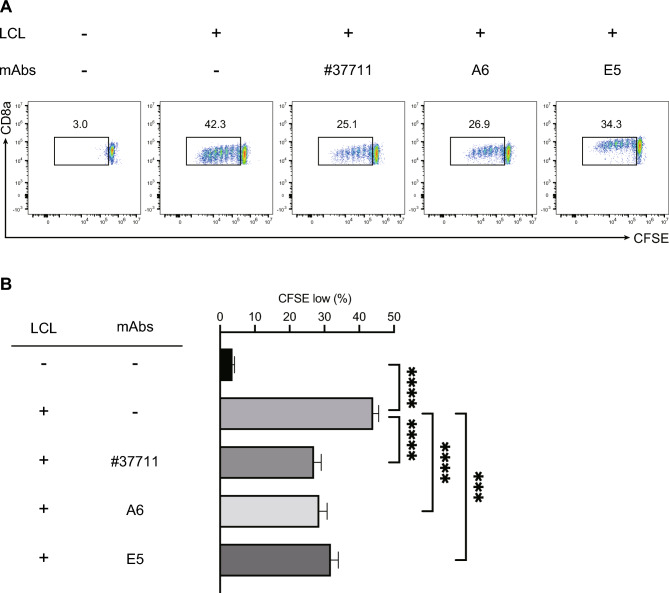
Anti-CD80 mAbs partially suppress CD8^+^ T cell proliferation in the presence of LCLs (**A**) Representative flow cytometry plots showing CD8a and CFSE in the blocking assay. CFSE-labeled CD8^+^ T cells were co-cultured with autologous LCLs for 120 hours in the presence or absence of mAbs. (**B**) Proportion of CFSE-low cells for the indicated conditions. Statistical data are presented as mean ± SD and were analyzed by one-way ANOVA with Bonferroni’s multiple comparisons test. ***p <0.001, ****p <0.0001. To match the isotype of anti-CD80 mAbs, mouse IgG1 antibodies were used in this experiment.

The Rtx-type amino acid substitutions in anti-CD80 IgG1 mAbs (E3R and E5R) had no impact on ADCC activity (Fig. 5A). In contrast, anti-CD80 IgG3 mAbs (A6G3^DLE^, E3G3^DLE^, and E5G3^DLE^) exhibited lower ADCC activity compared to their IgG1 counterparts, consistent with previous studies (Fig. 5C, Supple Fig. 6)^22^. Furthermore, to mimic the elimination of EBV-infected cells in patients, an ADCC assay was conducted using autologous lymphoblastoid cell lines (LCLs) and NK cells, which demonstrated comparable cytotoxic activity (Fig. 5D).

To further evaluate the potential of CD80 as a target antigen for ADCC against EBV⁺ lymphoma cells, we performed PBMC-mediated ADCC assays using Raji and Daudi cells with samples from six additional healthy donors. PBMCs from all donors showed significantly enhanced ADCC activity in the presence of anti-CD80 antibodies compared with the isotype control (Supplementary Fig. 5B–E), and this effect was dependent on antibody concentration (Supplementary Fig. 5F). Although killing efficiency varied among donors, it also differed according to target cell type and the antibody used, suggesting that both CD80 expression levels and epitope specificity contribute to the observed ADCC activity.

### T cell activation is maintained even at concentrations of anti-CD80 mAbs inducing ADCC activity

Finally, given the importance of maintaining T cell activity for antitumor immunity in cancer therapy, we investigated the effects of anti-CD80 mAbs on T cell activation. CD8^+^ T cells isolated from peripheral blood were labeled with CFSE and cocultured with autologous LCLs for 120 hours. Vigorous proliferation of CD8^+^ T cells was observed in the presence of LCLs but not in their absence. The addition of CD80 mAbs partially suppressed CD8^+^ T cell proliferation, but LCL-induced T cell activation was largely maintained (Fig. 6A and B).

In conclusion, analysis of the newly developed CD80-specific mAbs revealed that antibody-mediated cytotoxic effector functions depend on the type of antigen recognized by the variable region, rather than its constant region. The CD80-specific antibodies developed in this study effectively induced cytotoxicity against EBV-positive B cell lymphomas and EBV-immortalized B cells, while preserving T cell activation. These findings suggest that these antibodies may serve as promising therapeutic agents for EBV-associated diseases and CD80-expressing neoplastic disorders.

## Discussion

For antibody generation in this study, dimerized CD80-Fc was used for mouse immunization, while monomeric CD80-His was employed for FACS sorting of antigen-specific memory B cells. Although FACS sorting with CD80-Fc detected CD19^+^B220^+^IgG1^+^CD38^+^His-tag^+^ cells that appeared antigen-specific, PCR amplification was successful only for LCs and not for HCs (data not shown). This suggests that IgM or IgA memory B cells may have been detected due to non-specific binding between the mouse IgG1 Fc domain and Fc receptors on the cell surface.

Some clones bound to CD80-His in ELISA but failed to bind to the surface of Raji^WT^ cells. A possible explanation is that the epitope may be located in the inner region of CD80 when dimerized, rendering it inaccessible due to structural constraints. Additionally, CD80ECD-His was used as the antigen for FACS sorting and ELISA, suggesting the possibility that antibodies against the His-tag were selected, presenting a challenge with this approach. Enhancing antibody screening efficiency may be achieved by using dimeric antigens that mimic the *in vivo* structure for both FACS and ELISA. Practically, the three clones ultimately selected from 20 antigen-specific B cells exhibited very high affinity and are expected to be effective *in vivo*.

Possible reasons for the absence of CDC activity despite the presence of ADCC activity include glycosylation issues and steric hindrance affecting Fc-C1q binding. Furthermore, although antigen density and the ability of surface‑bound IgG to assemble into higher‑order oligomers are known determinants of CDC, this study did not evaluate the structural properties of CD80 or the mAbs that would influence such multimerization^23,24^. Although Fc mutations that retain ADCC but lack CDC activity are known, we used the same Fc domain as the common IgG1 allotype or rituximab, without introducing mutations that would impair CDC function^25^. Bivalent antigen binding may cause steric hindrance to C1q interaction, in which case univalent binding could improve geometrical alignment^26^, and similar improvements may be applicable to our antibodies. Furthermore, EGFR antibodies have shown enhanced CDC activity when combining two non-competitive antibodies, presumably by improving geometrical arrangement^27^. Three amino acid substitutions (S239D, A330L, I332E) in the IgG3 Fc domain are known to abolish CDC activity while enhancing ADCC^20^, which may explain the lack of CDC activity observed in the IgG3-type antibodies. In the ADCC assays performed in this study, purified NK cells were used as effector cells in order to clearly demonstrate antibody‑dependent cellular cytotoxicity mediated specifically by NK cells. While the use of PBMCs may better reflect physiological conditions by providing a heterogeneous effector cell population, employing purified NK cells allowed direct evaluation of NK cell‑mediated ADCC without confounding contributions from other immune cell types. Therefore, the present results establish the intrinsic ADCC capability of the developed antibodies, although further validation using PBMCs would be informative for assessing their activity under more physiological conditions.

Currently, no anti-CD80 antibodies are clinically available as monotherapy for antitumor activity. Galiximab, a primatized anti-CD80 mAb, was tested in combination with rituximab for recurrent follicular lymphoma. Although it was well tolerated and safe, it showed no efficacy^28^. Clinical trials involving Galiximab did not specifically target EBV-positive B-cell lymphomas, which may have limited its therapeutic effect due to low CD80 expression in tumor cells. As CD80 is a hematopoietic cell-specific molecule, unexpected side effects in other organs are less likely. Humanized anti-CD30 mAbs have not succeeded in treating HL^29^, suggesting that our antibodies may also be ineffective in their “naked” form. However, development of an antibody-drug conjugate, such as brentuximab vedotin, may overcome this limitation.

Regarding autoimmune diseases, CD80-high-expressing B cells have been identified in rheumatoid arthritis and multiple sclerosis^30,31^. Suppression of T-cell activation by inhibiting CD80-CD28 binding is useful for controlling autoimmune diseases, as evidenced by the clinical application of Abatacept, a CTLA4-Ig^32^. Although this study did not perform a detailed evaluation of blocking, it is possible that autoimmune diseases could be controlled by inhibiting CD80-CD28 binding or by selectively eliminating CD80-overexpressing cells through ADCC activity.

## Limitations of the study

This study has several limitations. First, although glycosylation of the Fc region is known to affect antibody functionality, it has not yet been analyzed in this study. Second, while CD80 is known to interact with CD28, CTLA-4, and PD-L1^33^, the blocking capacity of the antibodies developed in this study against each of these molecules has not been evaluated, nor has epitope mapping been performed. Additionally, blocking experiments using anti-CD80 mAbs in cocultures of LCLs and T cells were not compared with isotype controls, and the possibility of Fc domain-mediated growth inhibition has not been excluded.

This study did not evaluate EBV-positive primary B cell lymphoma including CD80 cell surface expression and ADCC activity. Comparing the Raji cells and LCLs used in the experiments with clinical samples may provide an opportunity to evaluate the validity of targeting CD80. In addition, a quantitative comparison of CD80 surface density with CD20 expression was not performed, as such analysis would require calibrated quantitative flow cytometry to determine absolute antigen density. Antibody-dependent cellular phagocytosis (ADCP) is an important function for therapeutic antibodies but was not evaluated in this study. Finally, *in vivo* experiments and studies involving antibody-drug conjugates have not yet been conducted.

## Materials and methods

### Ethics statement

Animal care and experiments were conducted in accordance with the international guidelines of the ARRIVE and the Japanese Association for Laboratory Animal Science (JALAS). This study was approved by the Ethics Review Committee for Animal Experimentation of Hiroshima University with the approval number: A24-61. Experiments involving samples from healthy volunteers were approved by the Institutional Review Board of Hiroshima University Graduate School of Biomedical and Medical Science. Informed consent was obtained from all healthy volunteers. This study was conducted in accordance with the Helsinki Declaration and approved by the Ethics Committee of Hiroshima University Graduate School of Biomedical and Medical Science.

### Acquiring microarray and RNA-seq data

RNA-seq data were retrieved from the Human Protein Atlas (HPA) (https://www.proteinatlas.org/). To identify differentially expressed genes (DEGs), group comparisons were performed using the edgeR R package, as previously described^34^. Membrane protein-encoding genes were searched in HPA database using terms as follows; (protein_class:Predicted membrane proteins AND ihc_ab_validation:Enhanced - Independent, Enhanced - Orthogonal, Supported, Approved AND uniprot_evidence:Evidence at protein level). To extract tumor-specific molecules, genes matching the criteria described below were removed; (“RNA tissue distribution” = “Detected in all” or “Detected in many”) OR (“RNA single nuclei brain distribution” = “Detected in all” or “Detected in many”)

### Mice

C57BL/6JJcl mice were purchased from CLEA Japan. Mice were bred and maintained under specific pathogen-free conditions. To harvest spleen cells, the mice were euthanized by cervical dislocation under isoflurane anesthesia.

### Production of recombinant CD80 and immunization

To produce recombinant human CD80-Fc protein, the human Ig leader sequence, CD80 extracellular domain (CD80ECD; amino acids Val35-Asn242), mouse IgG1 Fc domain, and 8xHistidine-tag were cloned into the pcDNA4 vector. For CD80-8xHistidine-tag (CD80-His) protein production, human Ig leader sequence, CD80ECD and 8xHistidine-tag were cloned into pcDNA4 vector (amino acid sequence listed in Table S2). Plasmid vectors were transfected into Expi293F cells using Expi293 Expression System Kit (Thermo Fisher Scientific). Expi293F cells were incubated 37℃, 8% CO_2_ incubator with shaking at 125 rpm (for 25 ml culture in a 200 ml flask) for 4-6 days. Supernatants were purified using Ni-NTA Agarose (QIAGEN) and dialyzed overnight in PBS. Protein purity was confirmed by SDS-PAGE, and concentrations were determined spectrophotometrically at 280 nm. C57BL/6JJcl female mice aged 8 weeks and weighing about 19 ± 2 g were immunized with alum-precipitated 100 µg CD80-Fc as in the previous study^18^.

### Preparation of single-cell suspension

Single-cell suspensions were prepared in Dulbecco’s modified Eagle’s medium (DMEM) supplemented with 1% fetal calf serum (FCS) and 1 mM EDTA as previously described^18^. To isolate mononuclear cells from spleens, tissues were mashed using frosted glass microscope slides. Single cell suspensions from spleen were resuspended in Gey’s solution (130 mM NH_4_Cl, 5.0 mM KCl, 0.8 mM Na_2_HPO_4_, 0.18 mM KH_2_PO_4_, 5.6 mM Glucose, 0.03 mM Phenol red, 1.0 mM MgCl_2_, 0.3 mM MgSO_4_, 1.5 mM CaCl_2_, 13 mM NaHCO_3_) for red blood cell lysis. Cell debris was removed using 70 µm nylon mesh. Cell pellets were resuspended in appropriate buffer.

### Flow cytometry for single-cell sorting

Single-cell sorting performed as previously described^18^. For single-cell sorting, spleen cells were treated with TruStain FcX™ (anti-mouse CD16/32) antibody (BioLegend, #101319) to reduce non-specific labeling of the cells. Cells were stained with anti-IgG1 (BioLegend, #406605) for 15 min on ice. After washing, cells were stained with CD80-His for 30 min on ice. After washing, cells were stained with His Tag-APC (BioLegend, #362605), CD19-BV605 (BioLegend, #115539), CD38-PE/Cy7 (BioLegend, #102717), and B220-APC/Cy7 (BioLegend, #103223) for 30 min on ice. The cells were resuspended in FACS buffer (PBS containing 1% FCS, 1 mM EDTA, and 0.05% NaN3) supplemented with 1 µg/ml propidium iodide (PI) to exclude dead cells. Single-cell sorting was performed on Special Order System FACSAria Fusion (BD Biosciences). Cells were directly sorted into 8-strip PCR tubes. Tubes containing single cells were stored at −80℃ until proceeding to RT-PCR. Flow cytometric data were acquired on CytoFLEX S (Beckman Coulter). Flow cytometric data were analyzed using FlowJo software (v10.8.1, BD Biosciences).

### Single-cell RT-PCR and monoclonal antibody production

Antibodies were identified and sequenced as described previously^18^. In brief, RNA from single cells was reverse-transcribed (PrimeScript™ II Reverse Transcriptase, Takara Bio) with random hexamer and oligo dT. For PCR amplification of HC and LC genes, semi-nested PCR was performed using PrimeSTAR^®^ HS DNA polymerase (Takara Bio), custom primers, and SimpliAmp Thermal Cycler (Applied Biosystems). PCR fragments were purified with FastGene™ Gel / PCR Extraction Kit (FastGene) and assembled into a linearized pcDNA vector using NEBuilder HiFi DNA Assembly Master Mix (New England Biolabs) according to the manufacturer’s instructions. Final 1 µl of assembled products mixed with 10 µl DH5α competent cell (Smobio) for transformation. The vectors were extracted and purified using FastGene Plasmid Mini Kit (FastGene) or FavorPrep Plasmid Extraction Midi Kit (Favorgen). HC and LC genes were cloned into each expression vector and sequenced using a CMV-forward primer (5’-CGCAAATGGGCGGTAGGCGTG-3’) located upstream of the cloning part. Monoclonal antibodies were produced and purified as previously described^18^.

### Plasmid

The human IgG1 Fc domain fragment was amplified from NCV2SG48 heavy chain expression vector using custom primers (Table S1)^19^. Rituximab-type mutants (R214K, V215A) were generated by overlap PCR using primers containing mutations. For improving the affinity for FcγRIIIa, three amino acid mutations (S239D, A330L, I332E) induced into human IgG3 Fc domain (IgG3^DLE^). The IgG3^DLE^ Fc domain fragment was synthesized commercially (gBlocks® Gene Fragments)(Table S1). pcDNA4 expression plasmids encoding IgG1 or IgG3 ^DLE^ Fc domain were generated by assembly of fragments using NEBuilder HiFi DNA Assembly Master Mix (New England Biolabs) according to the manufacturer’s instructions. To produce mouse-human chimeric antibody expression vector with the CD80-specific variable domain, the variable domains of three clones (A6, E3 and E5) were amplified using custom primers (Table S1), and assembled into linearized each Fc domain-containing vector using NEBuilder HiFi DNA Assembly Master Mix (New England Biolabs) according to the manufacturer’s instructions.

### ELISA

MaxiSorp ELISA plates (Thermo Fisher Scientific) were coated with 2 μg/ml purified CD80-His in 1xBBS (140 mM NaCl, 172 mM H3BO3, 28 mM NaOH) overnight at 4 °C, and then blocked with blocking buffer containing 1% BSA in PBS for 1 h. Antibodies diluted in PBS supplemented with 0.05% Tween 20 (PBS-T) were added and incubated for 2 h. HRP-conjugated antibodies were added and incubated for 2 h. Finally, wells were developed with the SureBlue™ TMB 1-Component Microwell Peroxidase Substrate (SeraCare) and stopped reaction with 1 M HCl, or ELISA POD Substrate TMB Kit (Nacalai) and stopped reaction with 1 M H_2_SO_4_. The absorbance at 450 nm was measured using iMark microplate absorbance reader (Bio-Rad). The absolute IC50 was calculated using a nonlinear regression curve fit on Prism (v10.0, GraphPad), according to the previously established protocol^18^.

### Affinity measurement using biolayer interferometry (BLI)

The binding affinity of obtained antibodies to CD80-His was examined by the BLItz system (Sartorius Japan) using Octet ProG Biosensors (Sartorius #18-5084) following the method of previous study^19^. 10 µg/ml of antibody was captured by the biosensor and equilibrated, followed by sequential binding of each concentration of CD80-His. For dissociation, biosensors were dipped in PBS-T for 300 sec. Results were analyzed on BLItz Pro (v1.3.1.3, Molecular Devices).

### Generation of CD80 knockout Raji cell (Raji^80KO^)

Guide RNAs targeting CD80 (5'-gctctgcgcccatctgacga-3') were cloned into px330-mcherry as previously described^35^. Plasmids were electroporated into wild-type Raji cells (Raji^WT^) using NEPA21 type II electroporator (Nepagene). Cells that had lost the expression of CD80 were sorted using cell sorter (FACSAria Fusion, BD Biosciences).

### Flow cytometry analysis for anti-CD80 mAbs

Raji^WT^ or Raji^80KO^ cells were treated with Human TruStain FcX™ (BioLegend, #422302) to reduce non-specific labeling of the cells. Subsequently, cells were stained with culture supernatants or purified mAbs. FITC anti-mouse IgG1 (BioLegend, #406605) and PE anti-human IgG Fc (BioLegend, #409303) were used as secondary antibodies. Mouse IgG1 UNLB (SouthernBiotech, #0102-01), Human IgG1 Kappa-UNLB (SouthernBiotech, # 0151K-01) and Human IgG3 Lambda-UNLB (SouthernBiotech, # 0153L-01) were used as isotype control. CD80-PE/Cy7 (BioLegend, #305217) and anti-Human B7-1/CD80 MAb (Clone 37711) (R&D Systems, #MAB140-SP) were used as a positive control.

### CDC assay

For human serum preparation, blood samples were collected in plain tubes (Venoject ® 2, VP-P070K, Terumo Corp) and kept at room temperature for 30 min. Serum samples were obtained after centrifugation at 1200 x g for 15 min. Raji cells were suspended in Opti-MEM (Gibco, #22600050) supplemented with 15% human serum or 6.25% Rabbit complement (Cedarlane Laboratories Ltd, # CL3305) and seeded into U-bottom 96-well plate (1 x 10^4^ cells/80 µl/well). 20 µl of serially diluted mAbs in Opti-MEM were added to each well and incubated at 37℃ for 4 hours. Rituximab (Selleck, #A2009) was used as a positive control. After incubation, 100 µL of FACS Buffer added to each well and centrifuged 450 x g for 1 min. Cells were suspended in FACS buffer supplemented with 1 µg/ml PI to analyze dead cells. The percentage of CDC activity was calculated by normalizing the cytotoxicity of each sample to that of the negative control and plotted against the concentration of mAbs.

### ADCC assay

Peripheral blood mononuclear cells (PBMCs) were isolated from buffy coats with density centrifugation using lymphocyte separation medium 1077 (Takara Bio, Shiga, Japan) from healthy donor. For NK cell purification, PBMCs were stained with biotinylated CD56 (BioLegend, #318320) for 15 min. Cells bound to biotinylated antibodies were captured using BD IMag™ Streptavidin Particles Plus (BD Biosciences) for 8 minutes, and the bounded fraction was collected. CD56 positive cells with at least 97% purity were used for subsequent cell culture experiments on the day. The lymphoblastoid cell line (LCL) was generated as previously described^36^.

For ADCC assay, Target cells (Raji^WT^, Raji^80KO^, and autologous LCL) were labelled with CFSE (Dojindo, #341-06443) before use, and suspended in RPMI-1640 supplemented with 10% FCS, 1% Penicillin-streptomycin. Target cells were seeded into U-bottom 96-well plates (3 x 10^5^ cells/50 µl/well) with 10 µg/ml of each mAbs. Rituximab (Selleck, #A2009) or Galiximab (MedChemExpress, HY-P99293) were used as positive controls. Human IgG1 Kappa-UNLB (SouthernBiotech, # 0151K-01) was used for isotype control. NK cells suspended in 50 µl were added at effector/target ratios of 10:1, 3:1, 1:1 (Final concentration of mAbs were 5 µg/ml). PBMCs suspended in 50 µl were added at effector/target ratios of 50:1. Plates were centrifuged at 20 x g for 2 min and incubated at 37℃ for 16 hours. After incubation, cells were centrifuged at 450 x g for 1 min and suspended in FACS buffer supplemented with 1 µg/ml PI. Percent-specific killing was calculated as follows; % specific killing = % PI positive target cells in cultures with both effectors and targets - % PI positive target cells in cultures with targets alone. For Supple Fig. 5B-F, percent-specific killing was calculated using the lowest concertation of isotype control as the baseline. For ADCC assays, donor samples were used as follows: Fig. 5 and Supple Fig. 5A, donor 1; Supple Fig. 5B-F, donors 2, 3, 4, 5, 6, and 7.

### T cell activation assay

CD8^+^ T cells were isolated from PBMCs using MojoSort™ Human CD8 T Cell Isolation Kit (BioLegend) according to the manufacturer’s instructions and labelled with CFSE (Dojindo). CD8^+^ T cells were co-cultured for 120 hours with 30 Gy-irradiated autologous LCLs at an effector-to-target ratios of 10:1 in the presence or absence of mAbs (10 µg/mL). LCLs were established as previously described^37^. In order to match the isotype of mAbs with anti-human B7-1/CD80 mAb (clone 37711, mouse IgG1), anti-CD80 mAbs of mouse IgG1-type shown in Fig. 3 were used in this experiment. Cells were treated with Human TruStain FcX™ (BioLegend, #422302) and subsequently stained with CD8a-APC (BioLegend, #300911).

### Statistics

Statistical analyses were performed using Prism (v10.0, GraphPad) software. Mann-Whitney *U* test was used for two-group comparisons. One-way ANOVA with Bonferroni’s or Dunnett’s multiple comparisons test were used for multi-group comparisons. *P*-value 0.05 was considered statistically significant. Asterisks indicate statistical significance for *p*-values: < 0.05 (single), < 0.01 (double), < 0.001 (triple), and < 0.0001 (quadruple).

## Supplementary Information

Supplementary Information 1.

Supplementary Information 2.

Supplementary Information 3.

Supplementary Information 4.

Supplementary Information 5.

## Data Availability

All data reported in this paper will be shared by the lead contact upon request. Any additional information required to reanalyze the data reported in this paper is available from the lead contact upon request.
