# Supplementary material for: CD80 (B7-1) as a potential therapeutic target in Epstein–Barr virus‑associated B cell diseases
Source: Sci Rep. 2026 May 26;16:24036. doi: 10.1038/s41598-026-55043-5 (PMC13438696; doi:10.1038/s41598-026-55043-5)
Supplement: Supplementary file 1 [file 41598_2026_55043_MOESM2_ESM.pdf]

Yoshizato et al.
Supplemental Fig. 1

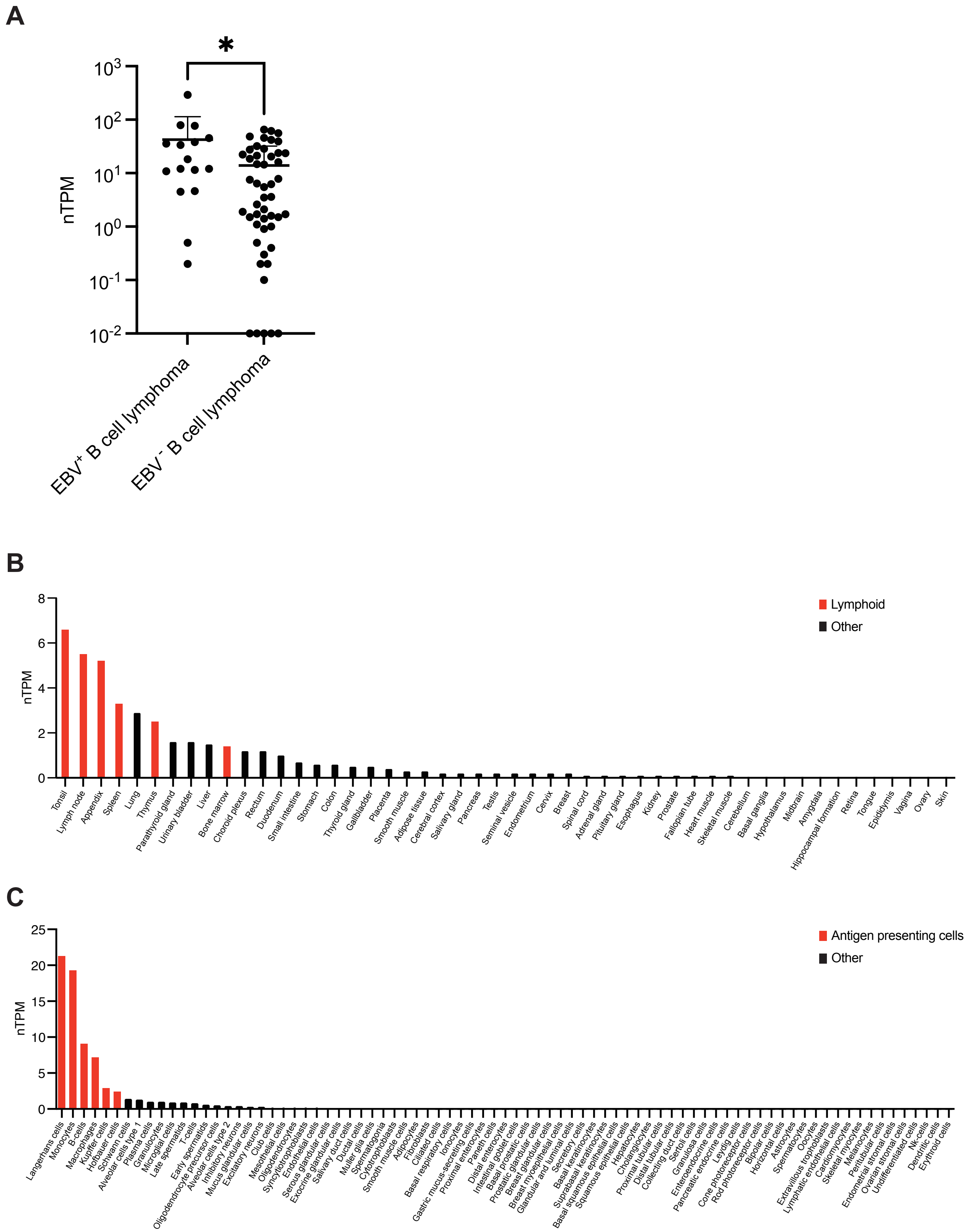

Yoshizato et al.
Supplemental Fig. 2

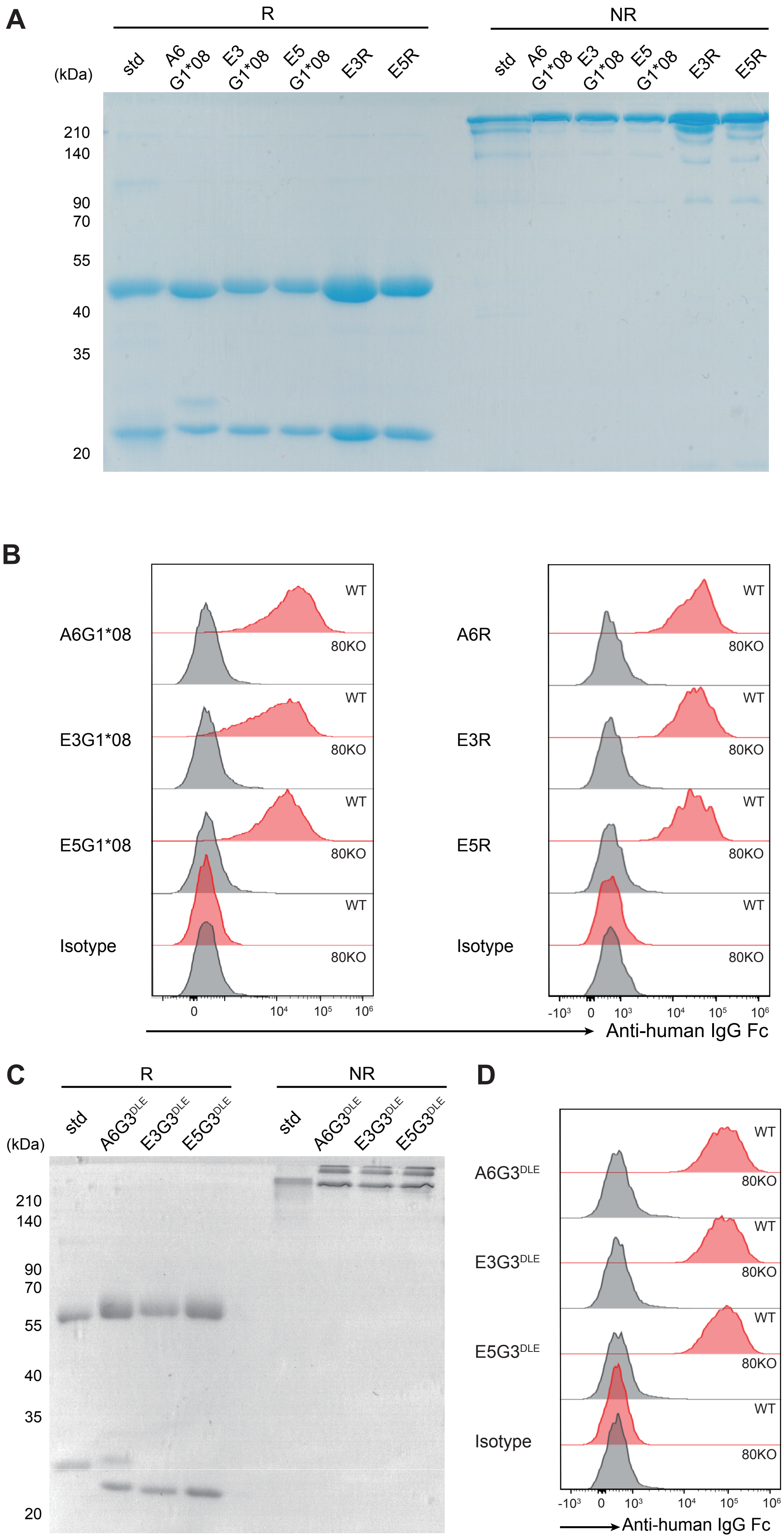

Yoshizato et al.
Supplemental Fig. 3

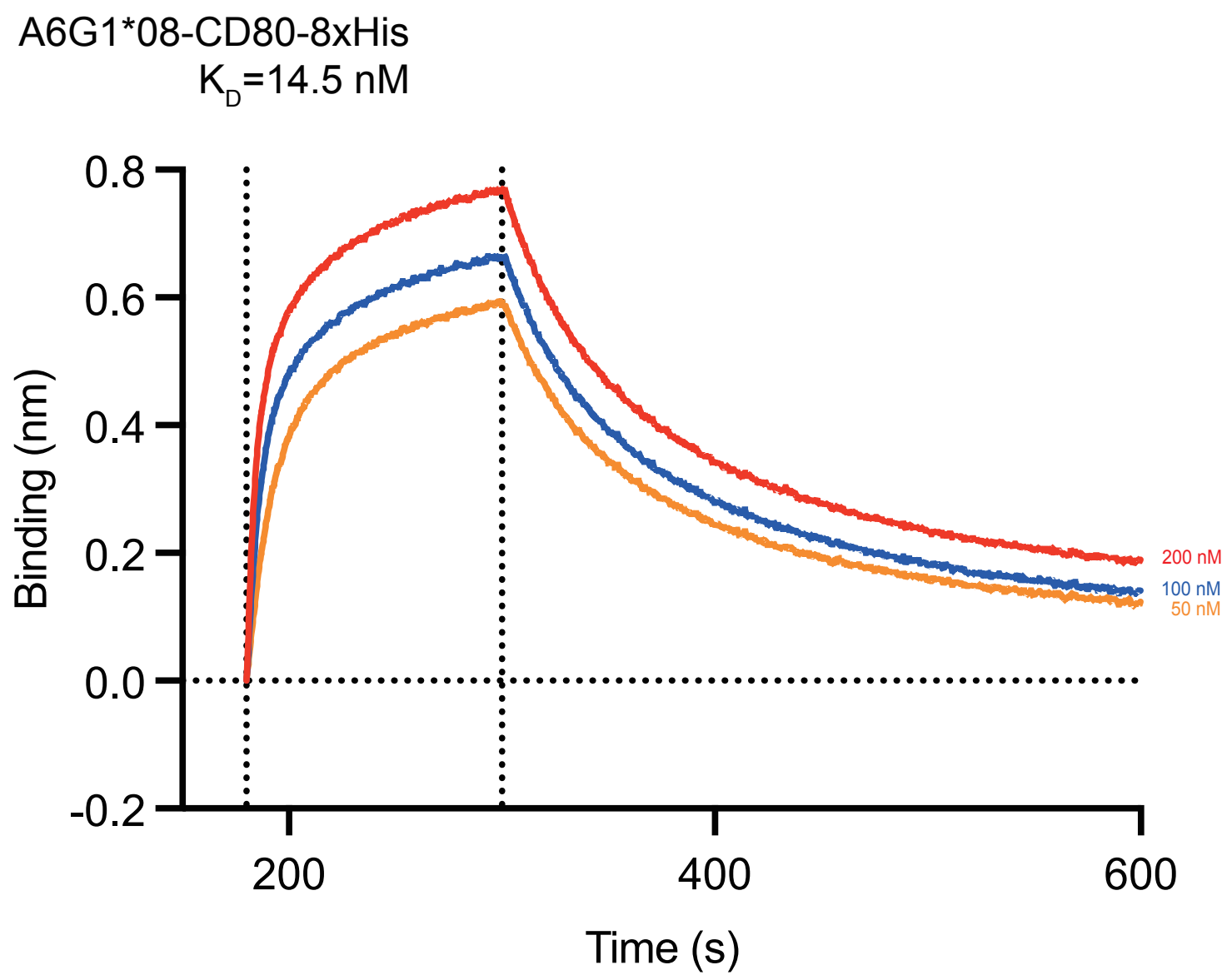

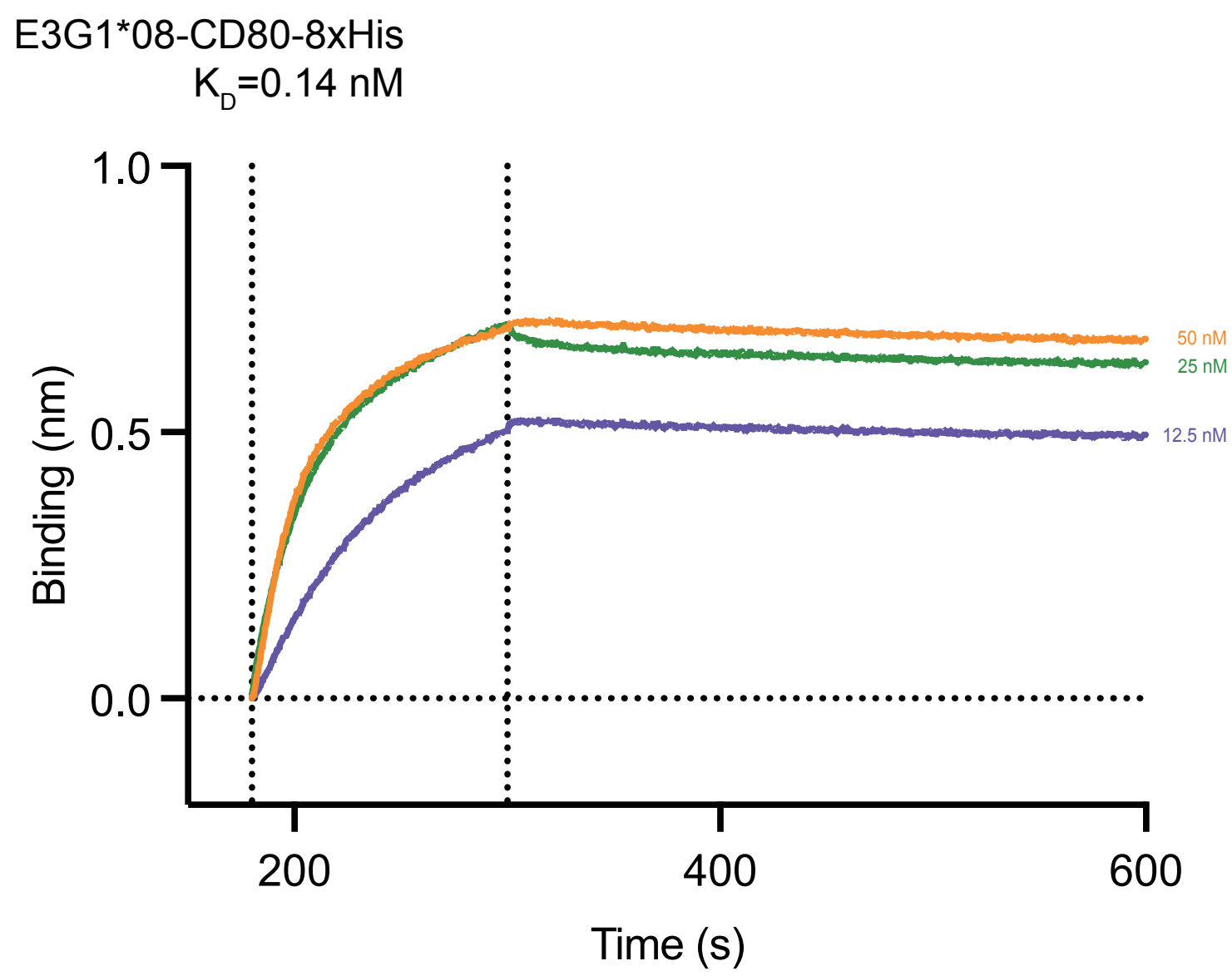

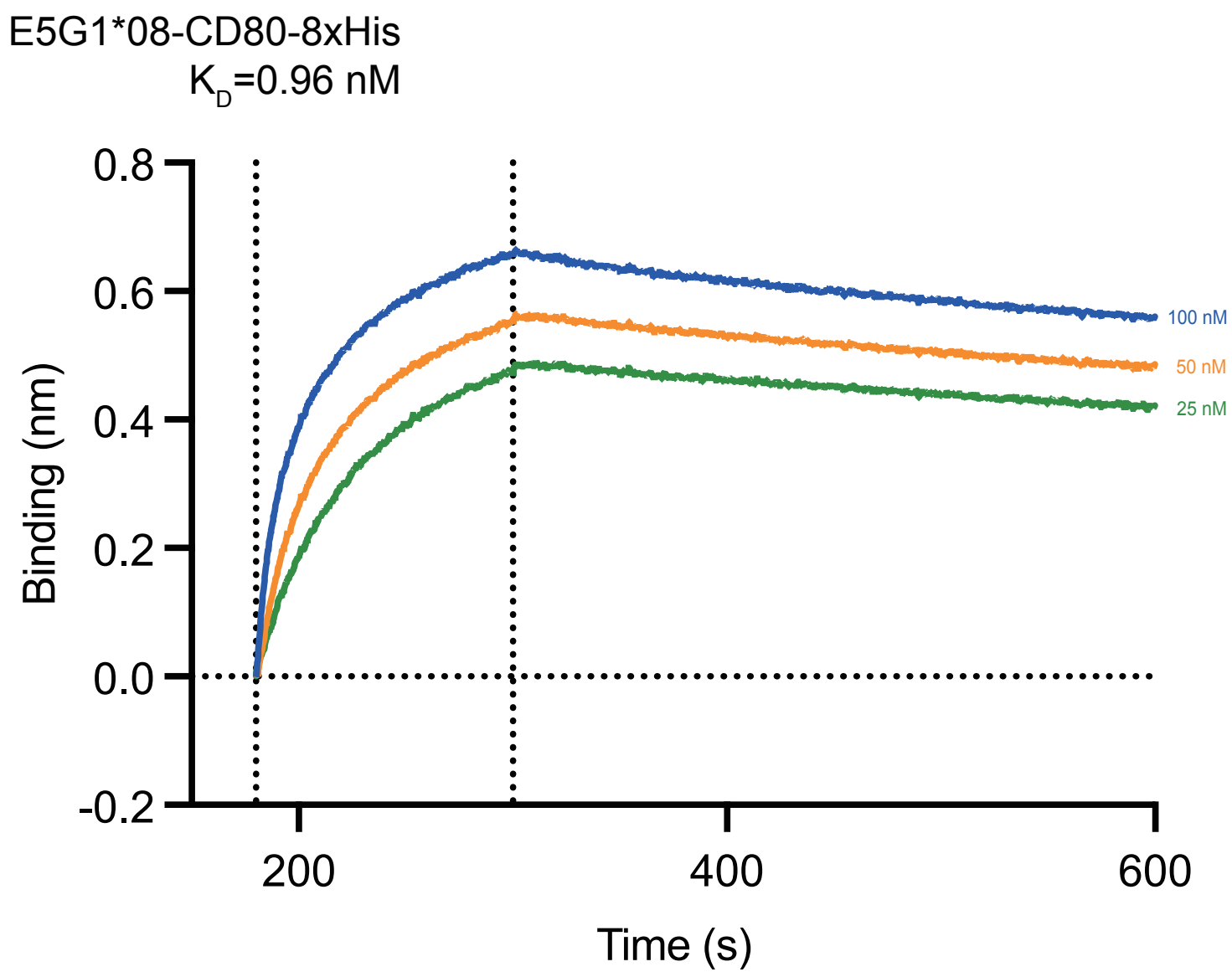

mAb	KD (nM)	ka (1/Ms) $x10^5$	kd (1/s) $x10^{-4}$
A6G1*08	14.5	6.3	91
E3G1*08	0.14	9.1	1.3
E5G1*08	0.96	4.9	4.7

Yoshizato et al.
Supplemental Fig. 4

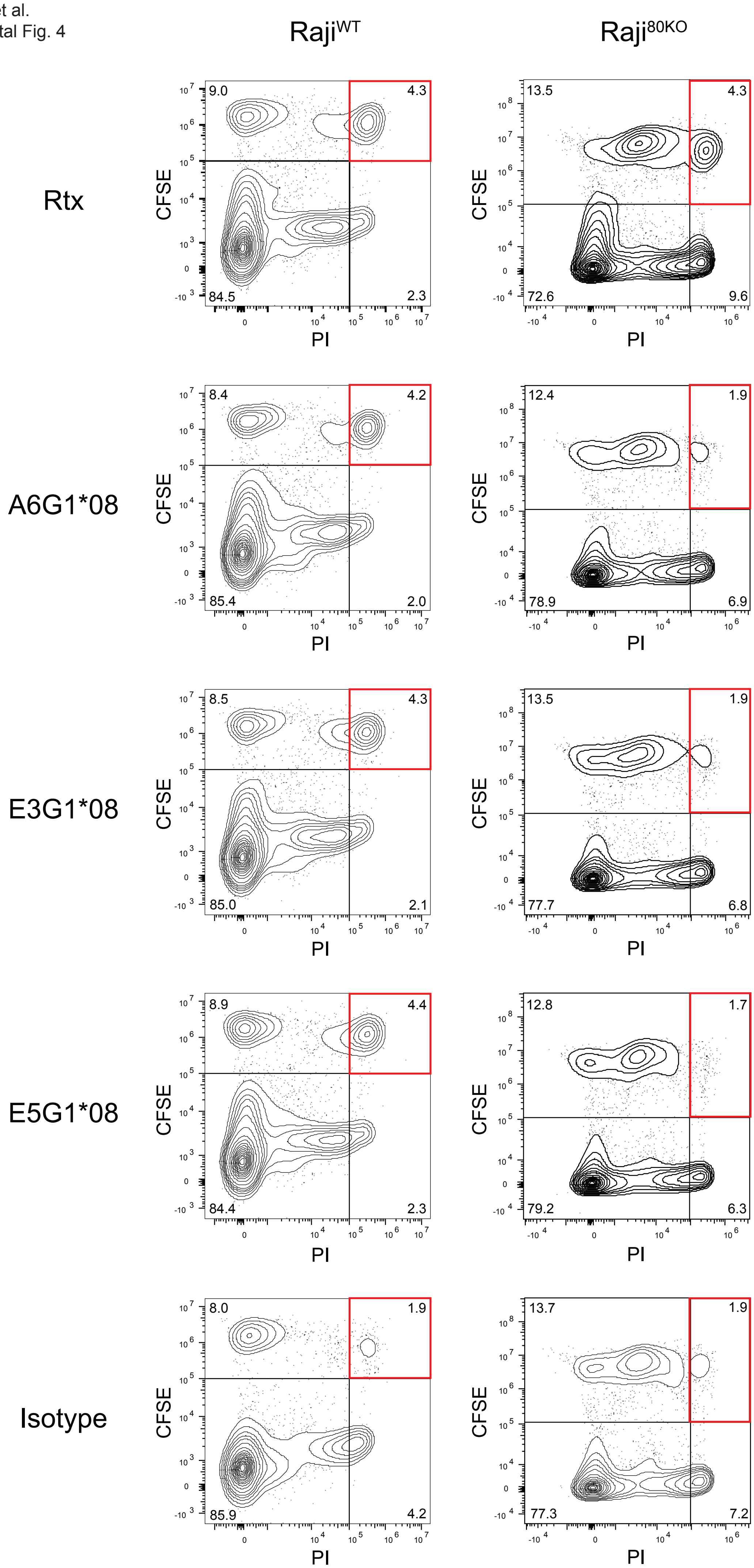

A

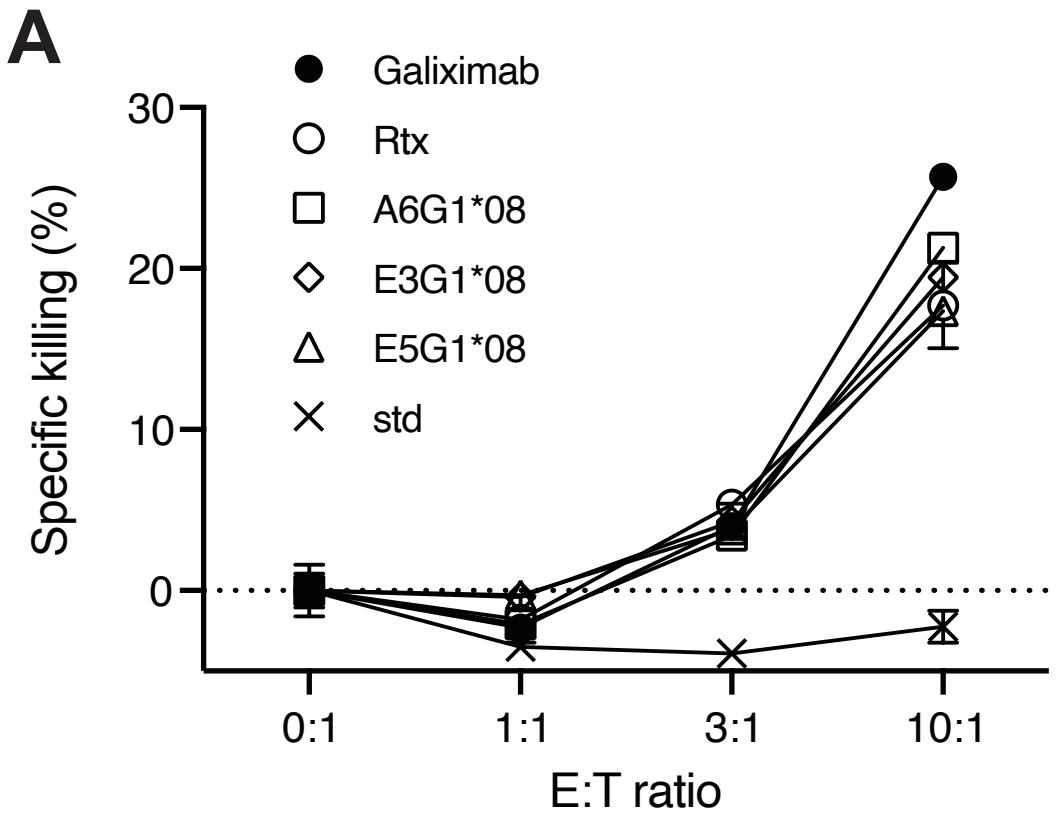

B

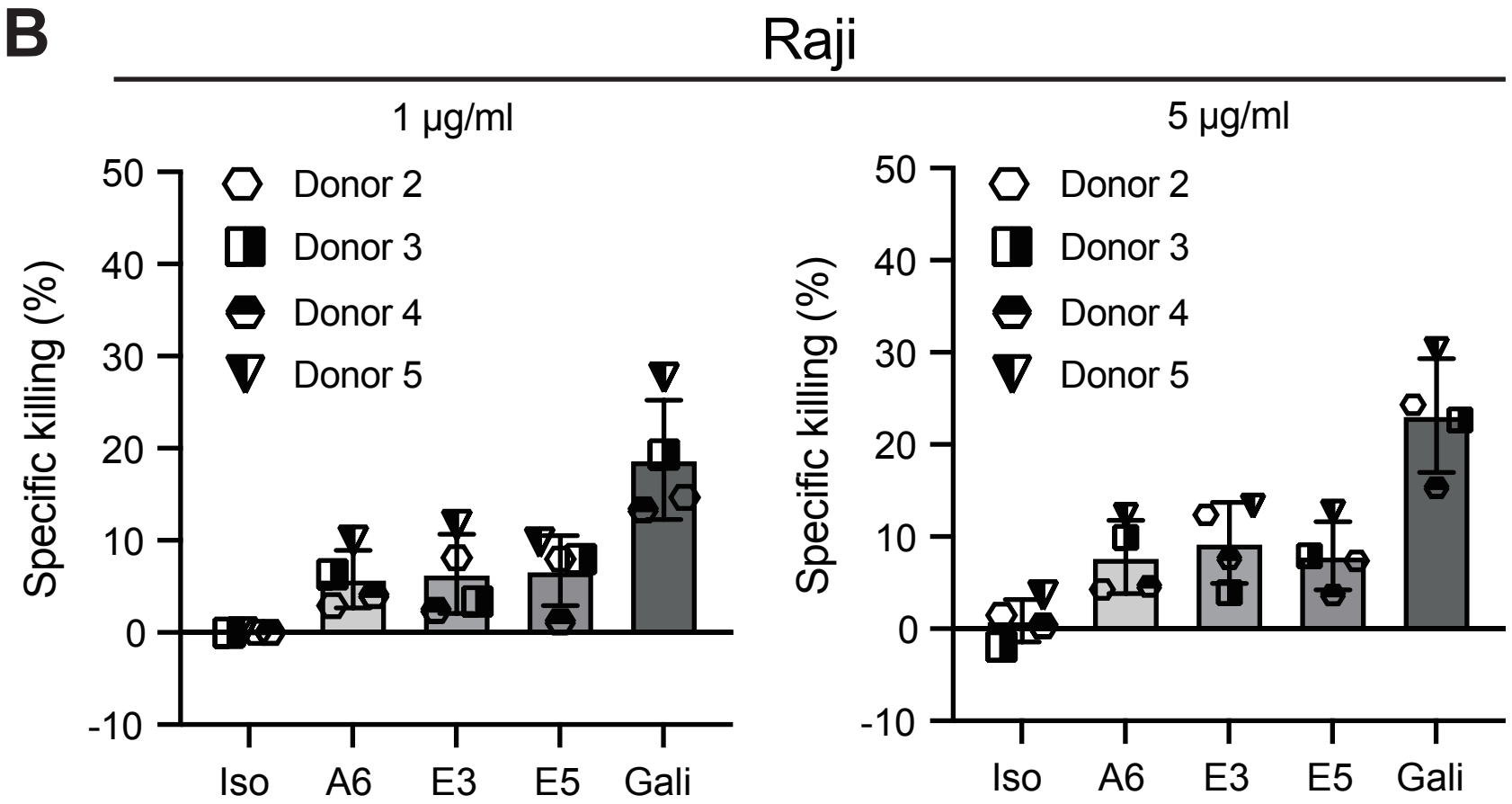

C

ADCC against Raji cells

PBMC	Ab conc. (μg/ml)	A6	E3	E5	Gali
Donor 2	1	ns	***	***	***
	5	ns	**	ns	***
Donor 3	1	*	ns	**	***
	5	**	ns	**	***
Donor 4	1	ns	ns	ns	***
	5	*	**	ns	***
Donor 5	1	***	***	***	***
	5	***	***	***	***

D

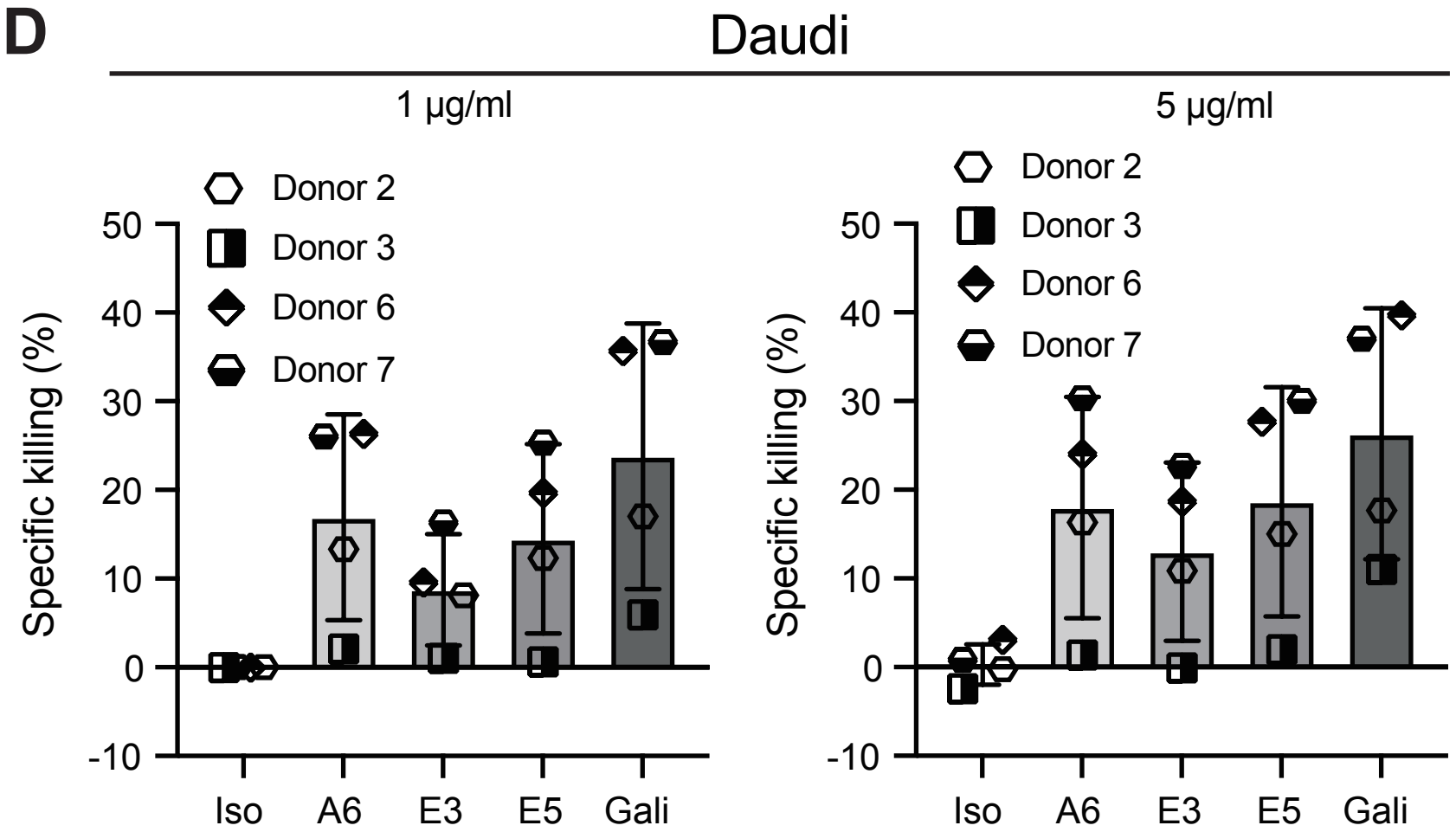

E

ADCC against Daudi cells

PBMC	Ab conc. (μg/ml)	A6	E3	E5	Gali
Donor 2	1	***	**	***	***
	5	***	***	***	***
Donor 3	1	ns	ns	ns	**
	5	*	ns	*	***
Donor 6	1	**	ns	*	***
	5	***	***	***	***
Donor 7	1	***	***	***	***
	5	***	***	***	***

F

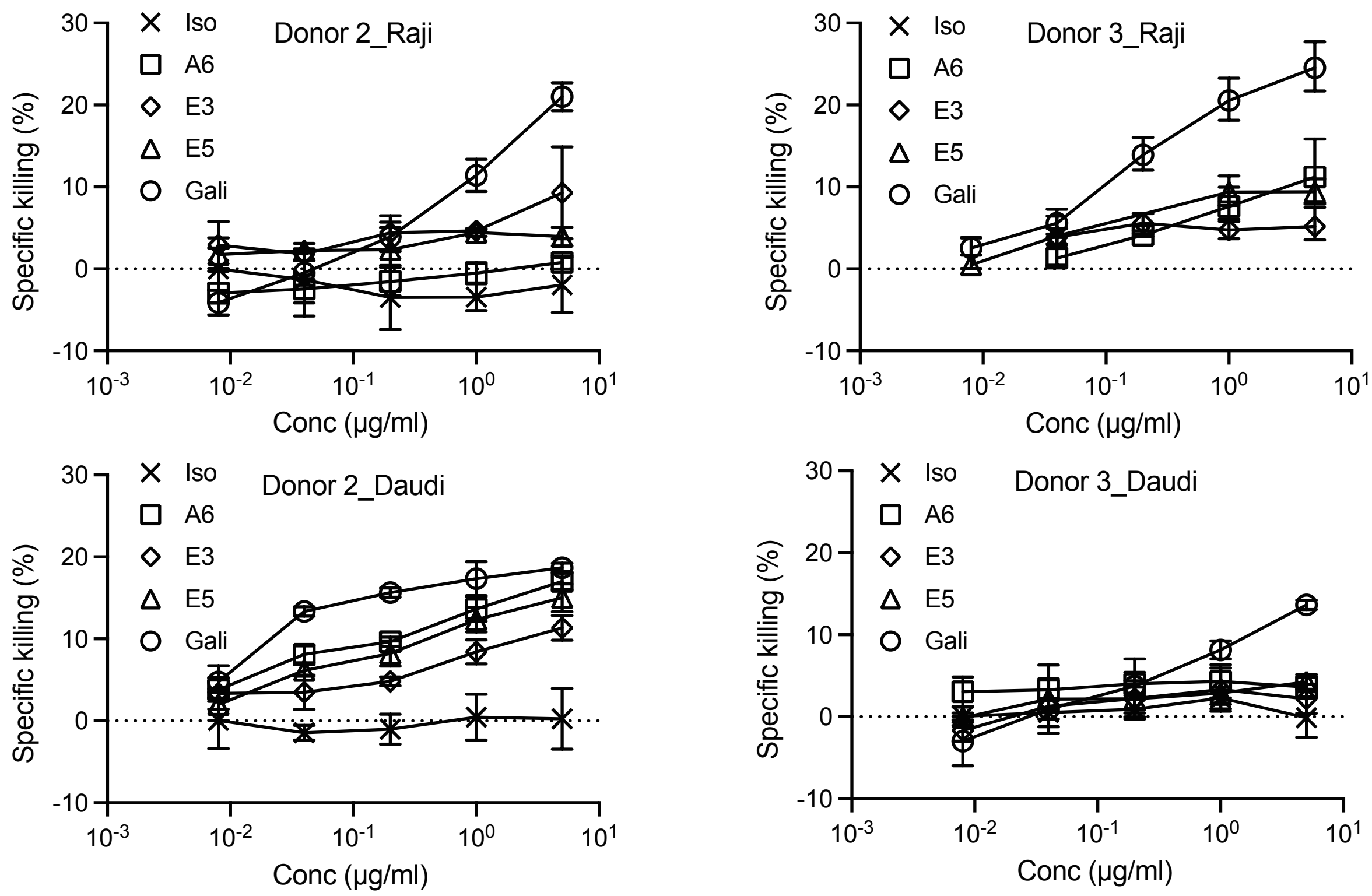

Yoshizato et al.
Supplemental Fig. 6

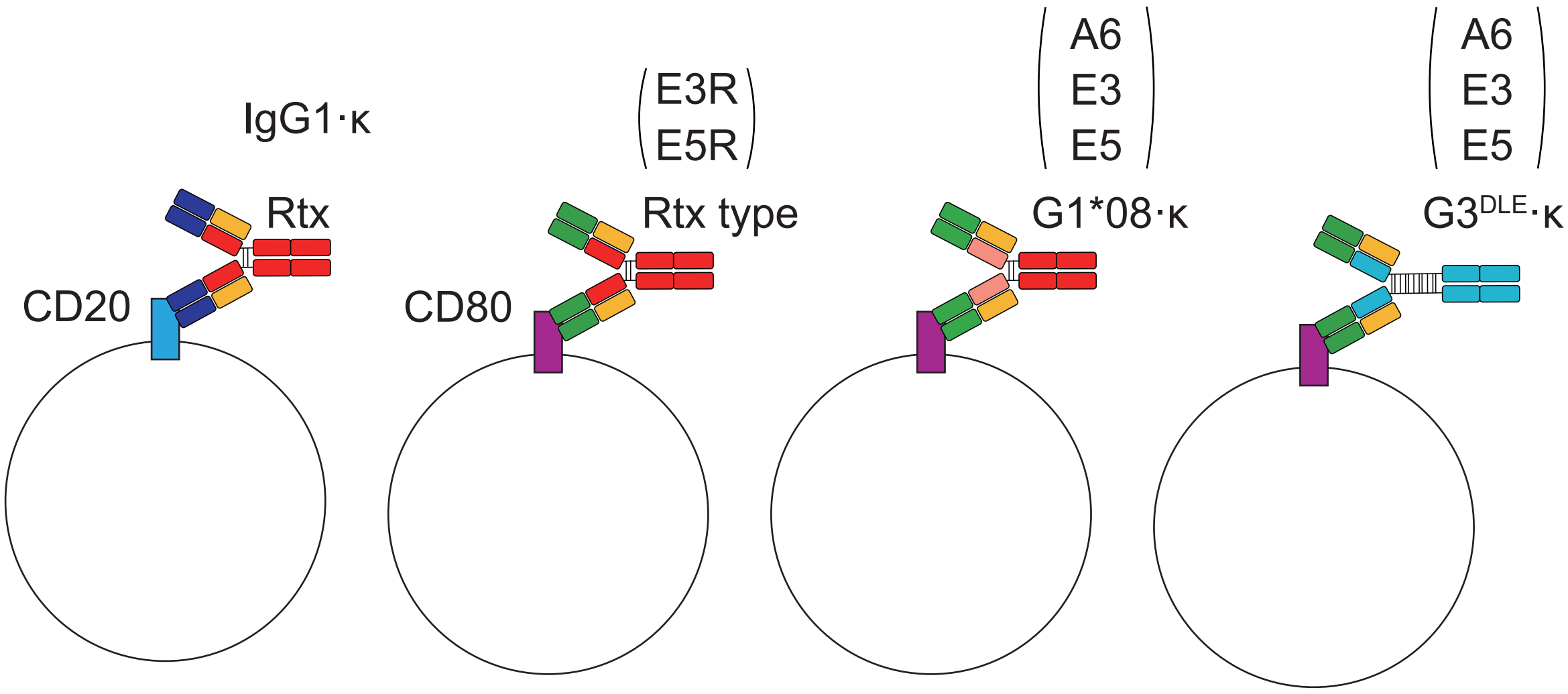

ADCC	++	+++	++ ~ +++	+/-
CDC	+++	-	-	-
