# Supplementary material for: CD80 (B7-1) as a potential therapeutic target in Epstein–Barr virus‑associated B cell diseases
Source: Sci Rep. 2026 May 26;16:24036. doi: 10.1038/s41598-026-55043-5 (PMC13438696; doi:10.1038/s41598-026-55043-5)

Supplemental figure A_uncropped

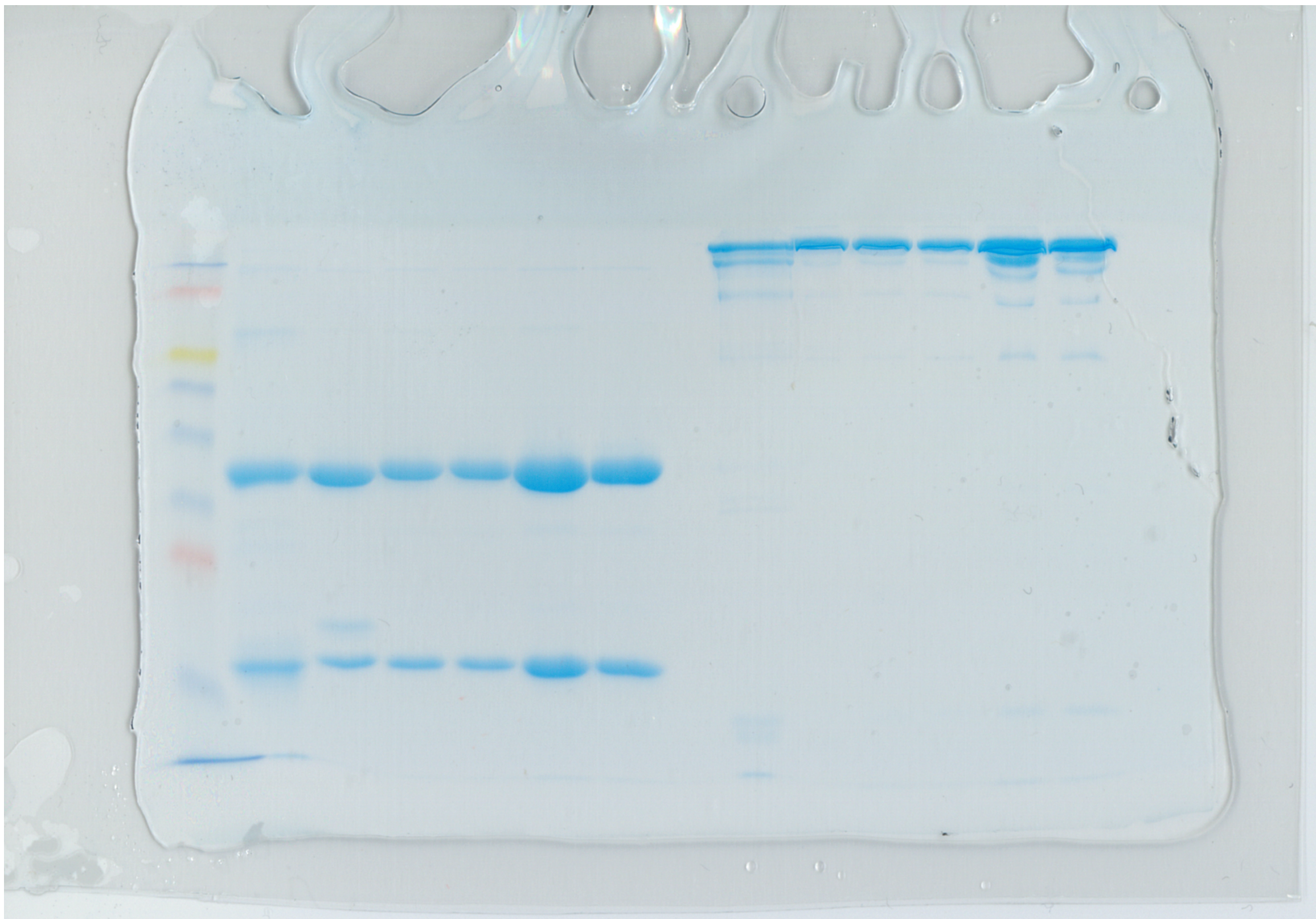

Supplemental figure C_uncropped

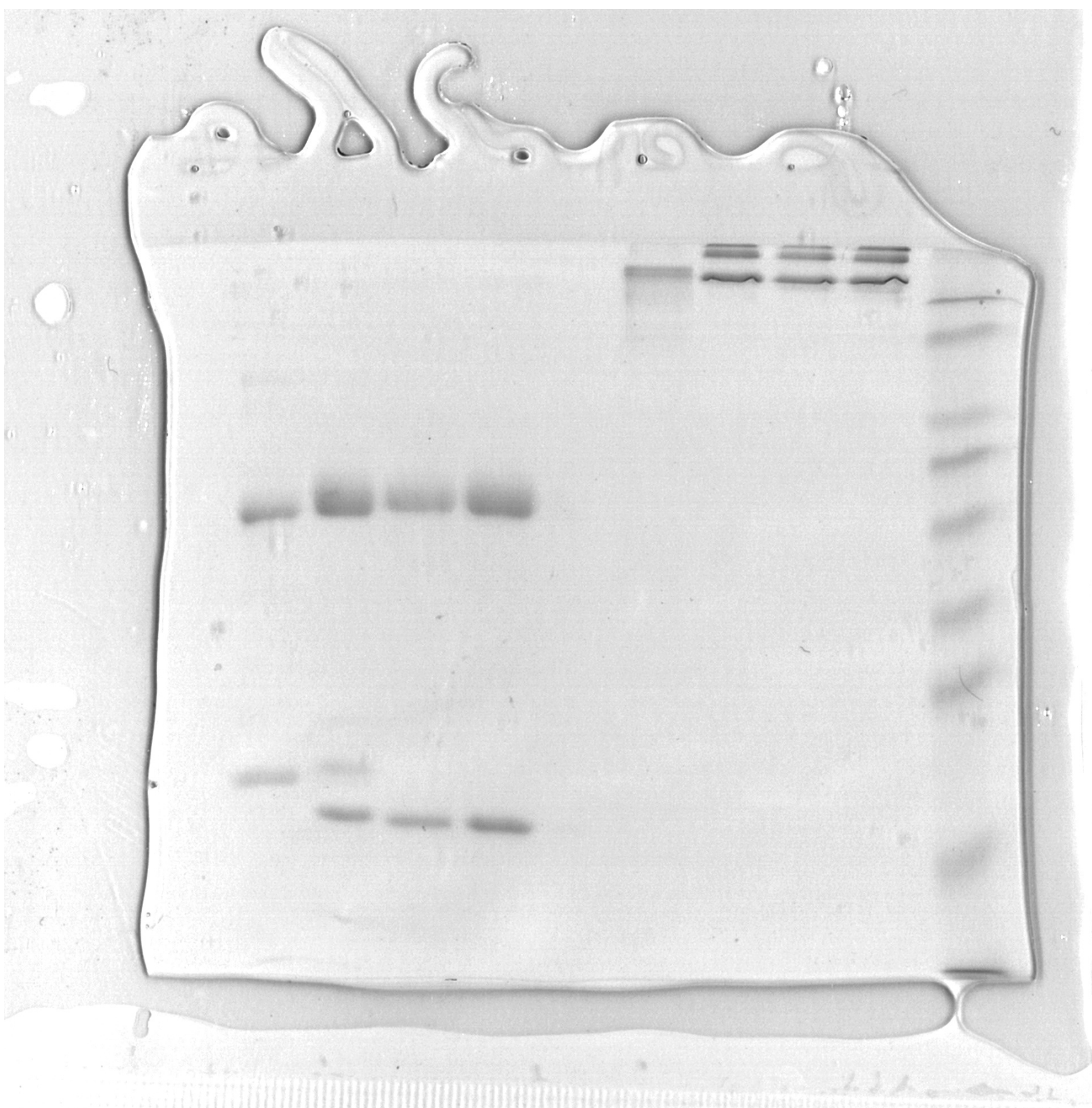

Supplement: Supplementary file 2 [file 41598_2026_55043_MOESM3_ESM.pdf]
