# Supplementary material for: CD80 (B7-1) as a potential therapeutic target in Epstein–Barr virus‑associated B cell diseases
Source: Sci Rep. 2026 May 26;16:24036. doi: 10.1038/s41598-026-55043-5 (PMC13438696; doi:10.1038/s41598-026-55043-5)
Supplement: Supplementary file 3 [file 41598_2026_55043_MOESM1_ESM.docx]

**Supplemental Information**

Document S1: Table S1 and S2

**Table S1.** Excel file containing primer sequences used for expression vector construction and variable domain cloning.

**Table S2.** Excel file containing protein sequences used for immunization and antigen-specific memory B cell sorting.

Document S2: Figures S1-S5

**Figure S1. CD80 expression in various cell lines**

(A) CD80 expression levels in 16 EBV-positive cell line datasets and 49 EBV-negative cell line datasets. EBV+, EBV-positive; EBV-, EBV-negative.

(B, C) CD80 expression levels shown as normalized TPM values. Data are retrieved from the Human Protein Atlas in different categories of (B) organs and (C) cell types. Lymphoid organs (B) and antigen presenting cells (C) are shown by red bars. (p13, lines 339-342)

**Figure S2. Establishment of IgG1 and IgG3-type chimeric anti-CD80 antibodies**

(A) SDS-PAGE image of monoclonal antibodies. Three micrograms of purified IGHG1*08-type (A6G1*08, E3G1*08 and E5G1*08) and rituximab-type (A6R, E3R and E5R) chimeric antibodies were loaded per well and separated on 10% SDS-PAGE under non-reducing (NR) or reducing (R) conditions. std, Human IgG1 Kappa-UNLB (SouthernBiotech, # 0151K-01).

(B) Evaluation of cell surface binding by CD80-specific IGHG1*08-type (A6G1*08, E3G1*08 and E5G1*08) and rituximab-type (A6R, E3R and E5R) chimeric antibodies. PE anti-human IgG Fc (BioLegend, #409303) was used as the secondary antibody. WT, wild-type Raji cells; CD80KO, CD80-knockout Raji cells; Isotype, Human IgG1 Kappa-UNLB (SouthernBiotech, # 0151K-01).

(C) SDS-PAGE analysis of mAbs. Three micrograms of purified IgG3^DLE^-type (A6G3^DLE^, E3G3^DLE^ and E5G3^DLE^) chimeric antibodies were loaded per well and separated on 10% SDS-PAGE under NR or R conditions. std, Human IgG3 Lambda-UNLB (SouthernBiotech, # 0153L-01).

(D) Evaluation of cell surface binding by CD80-specific IgG3^DLE^-type (A6G3^DLE^, E3G3^DLE^ and E5G3^DLE^) chimeric antibodies. PE anti-human IgG Fc (BioLegend, #409303) was used as the secondary antibody. WT, wild-type Raji cells; CD80KO, CD80-knockout Raji cells; Isotype, Human IgG3 Lambda-UNLB (SouthernBiotech, # 0153L-01).

**Figure S3. Affinity of chimeric antibodies to CD80-His protein**

Biolayer interferometry results showing the binding affinity of A6G1*08, E3G1*08, and E5G1*08 to CD80-His protein. Binding kinetics were measured at three antigen concentrations and analyzed using a 1:1 binding model.

**Figure S4. ADCC assay with Raji^WT^ and Raji^80KO^ cells**

Representative flow cytometry plots showing propidium iodide (PI) and CFSE (Dojindo, #341-06443) staining in ADCC assays using Raji^WT^ and Raji^80KO^ cells. PI^+^CFSE^+^ cells were defined as dead target cells (*red square*). Rtx, rituximab; Isotype, Human IgG1 Kappa-UNLB (SouthernBiotech, # 0151K-01).

**Figure S5. Anti-CD80 antibody-mediated ADCC against EBV^+^ lymphoma cell lines**

(A) NK cell-mediated specific killing at the indicated effector-to-target (E:T) ratios against Raji cells using IGHG1*08-type (A6G1*08, E3G1*08 and E5G1*08) chimeric antibodies, rituximab, or galiximab. Rtx, rituximab; Isotype, Human IgG1/κ. Data represent two independent experiments and are shown as the mean of duplicate technical measurements with error bars indicating the range.

(B-F) PBMC-mediated specific killing against EBV^+^ lymphoma cells using isotype (Iso), IGHG1*08-type (A6, E3 and E5) chimeric antibodies and galiximab (Gali).

(B and D) PBMC-mediated specific killing of Raji (B) and Daudi (D) cells at an E:T ratio of 50:1 using the indicated mAbs and concentrations. Each dot represents one individual, shown as the mean of technical triplicates. Data are presented as mean ± SD.

(C and E) Statistical analysis of specific killing in panels B and D was performed between anti-CD80 mAbs and isotype control using one-way ANOVA followed by Dunnett's multiple comparisons test. ns, not significant; **p* <0.05; ***p* <0.01; ****p* <0.001.

(F) PBMC-mediated specific killing of indicated cells at an E:T ratio of 50:1 using the indicated mAbs and concentrations. Data are shown as the mean of technical triplicates and presented as mean ± SD.

**Figure S6. Schematic summary of the present study**

IgG1*08-type and Rtx-type anti-CD80 mAbs exhibited ADCC activity comparable to rituximab. Switching to IgG3-type resulted in reduced ADCC activity. Anti-CD80 antibodies with three different Fc domains showed no CDC activity. G3^DLE^, IgG3^DLE^-type (A6G3^DLE^, E3G3^DLE^, and E5G3^DLE^)
